# Supplementary material for: Follicular Fluid Components in Reduced Ovarian Reserve, Endometriosis, and Idiopathic Infertility
Source: Int J Mol Sci. 2023 Jan 30;24(3):2589. doi: 10.3390/ijms24032589 (PMC9916781; doi:10.3390/ijms24032589)

Scatter plots of pairs of variables giving rise to Pearson correlation coefficient significantly different from zero considering the complete dataset

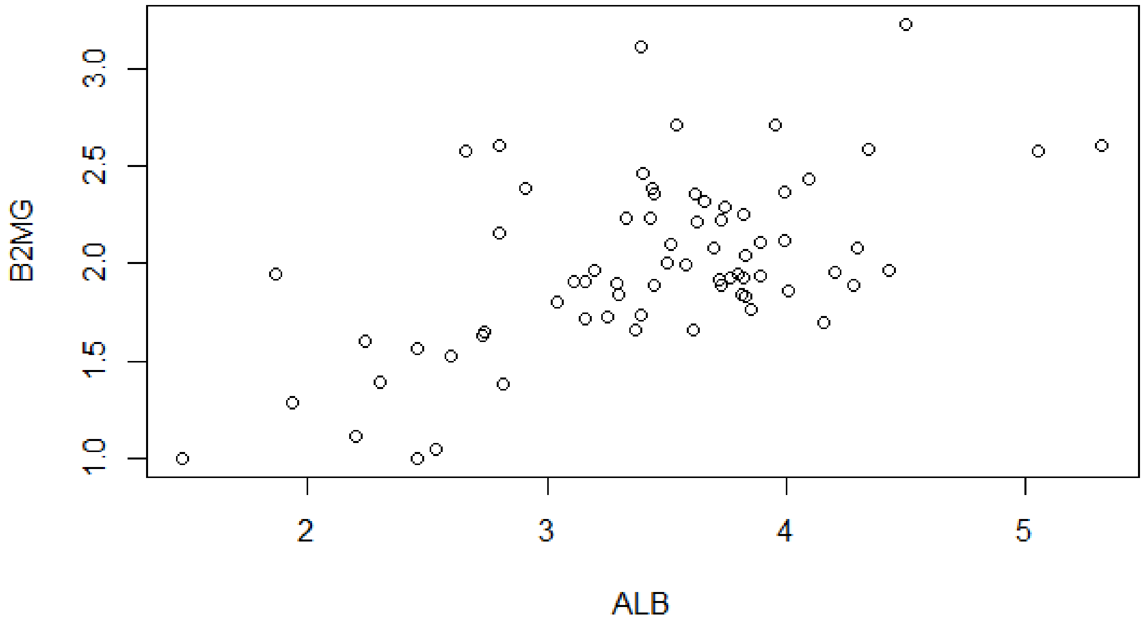

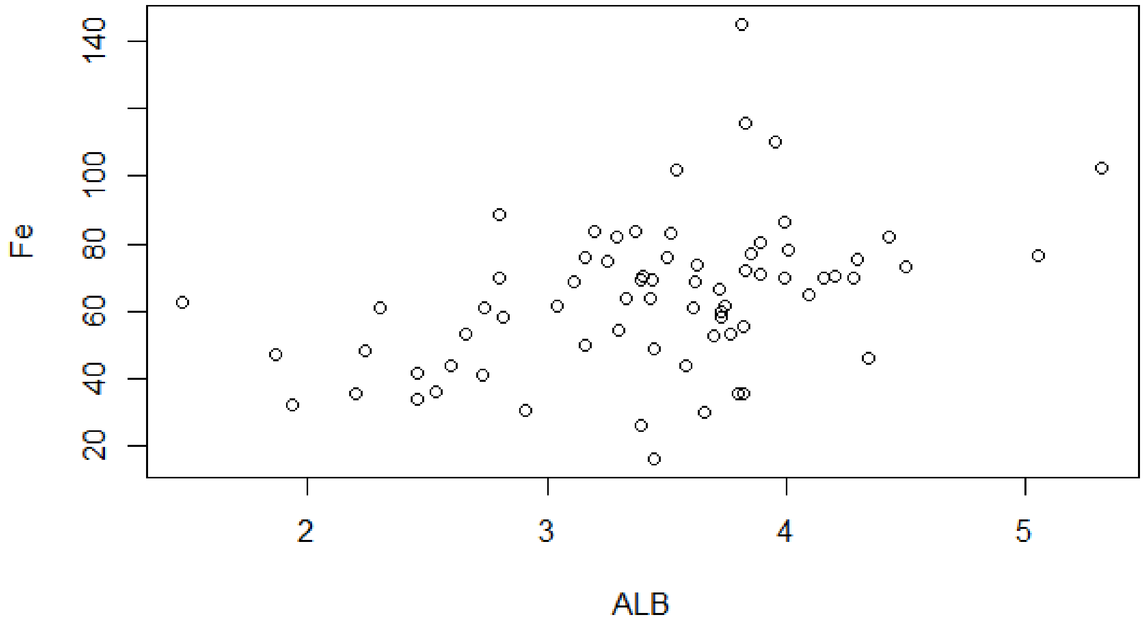

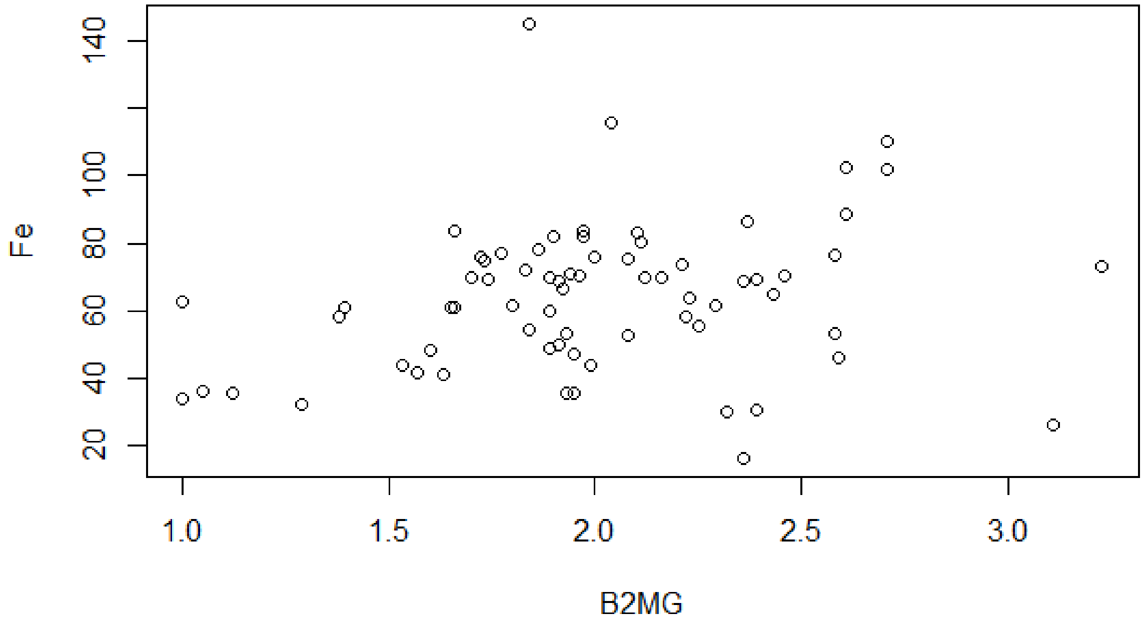
Fe
20
40
60
80
100
140
1.0
1.5
2.0
2.5
3.0
B2MG

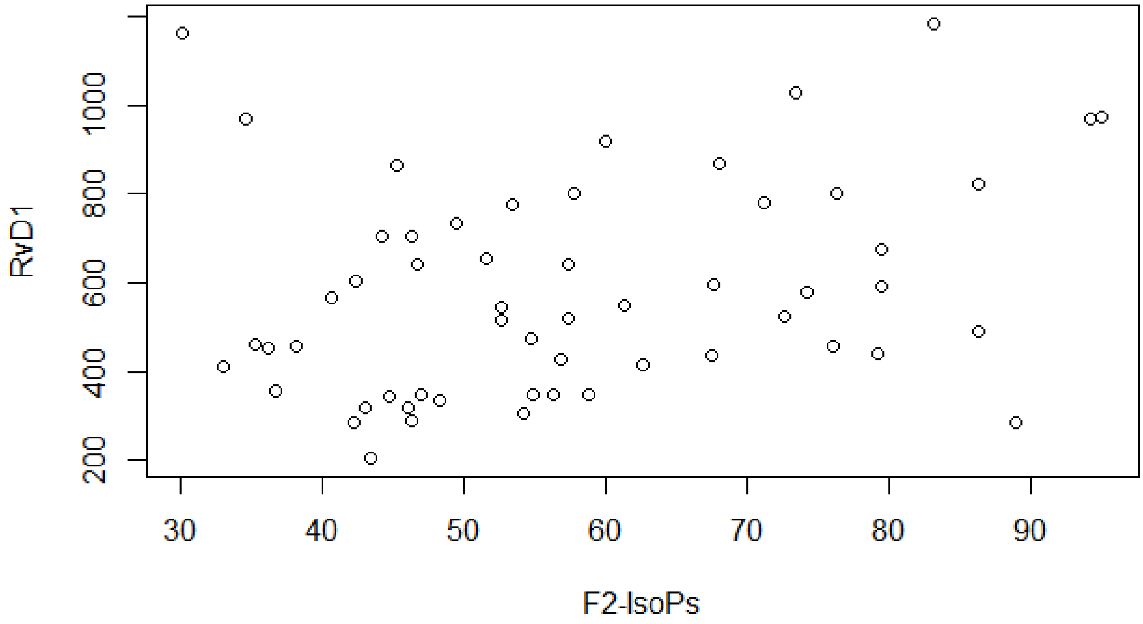
RvD1
200
400
600
800
1000
30
40
50
60
70
80
90
F2-IsoPs

Scatter plots of pairs of variables giving rise to Pearson correlation coefficient significantly different from zero considering the group of women with age greater or equal 38

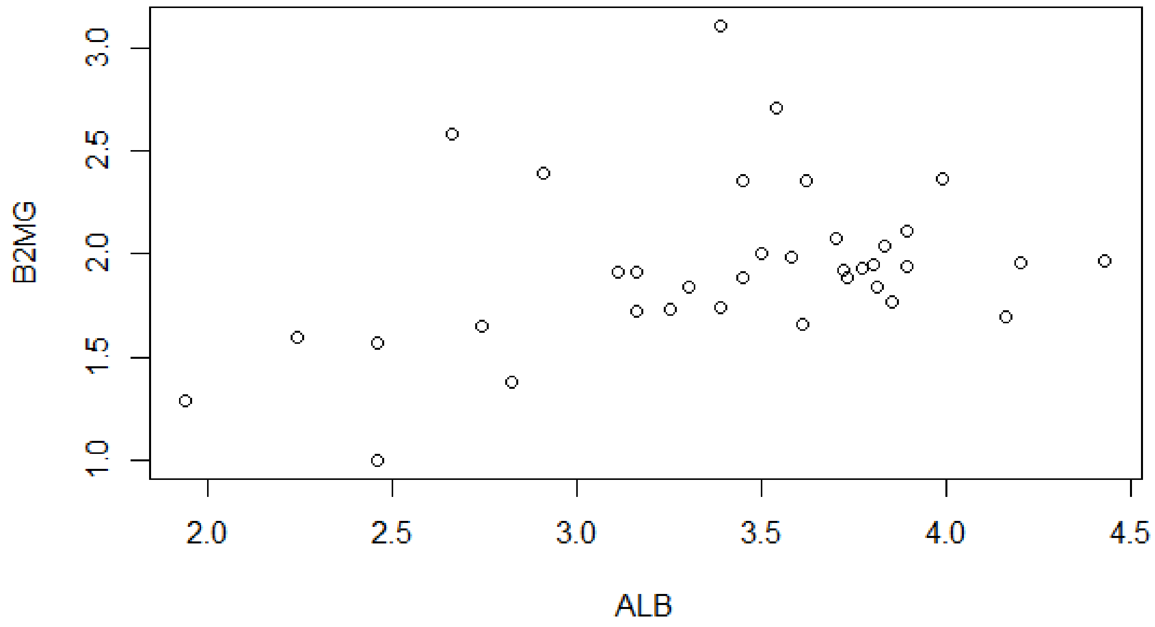

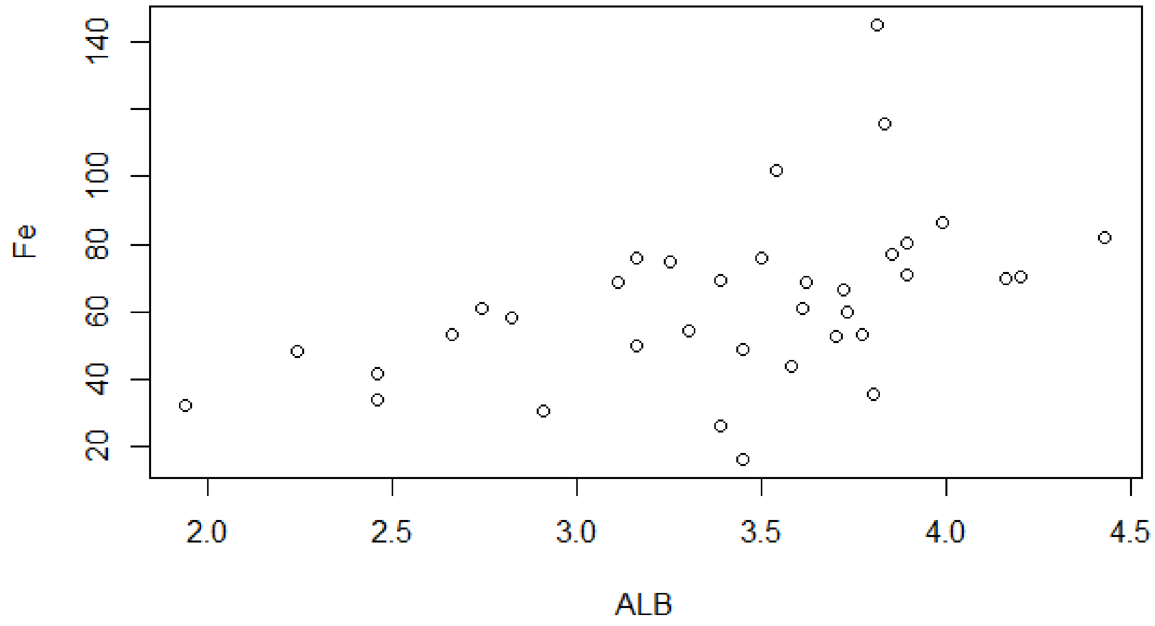

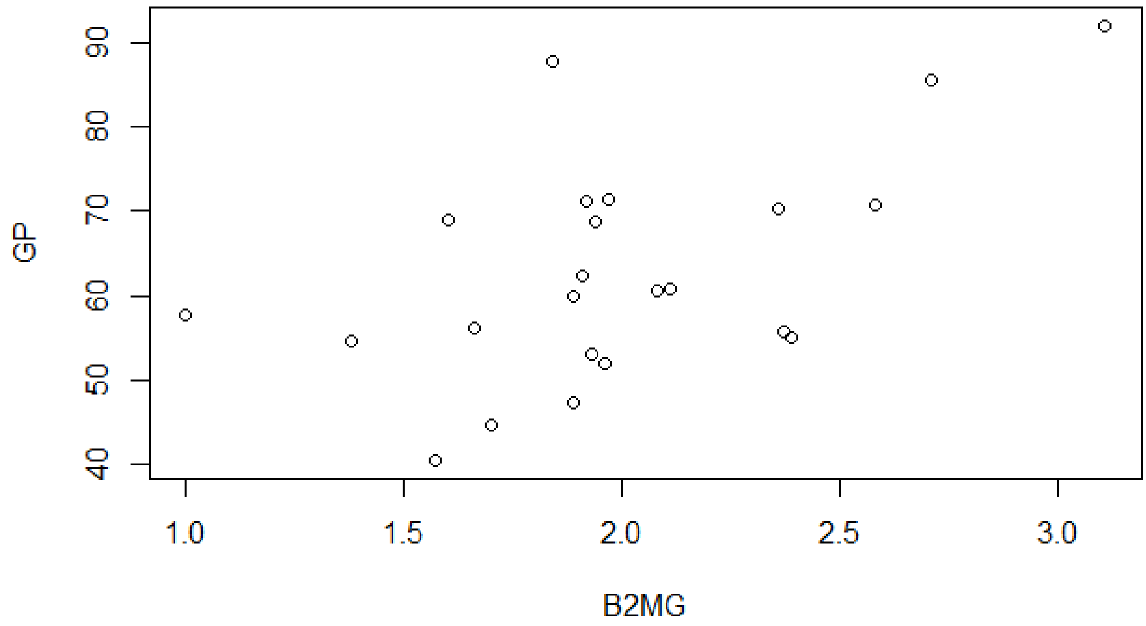
GP
40
50
60
70
80
90
1.0
1.5
2.0
2.5
3.0
B2MG

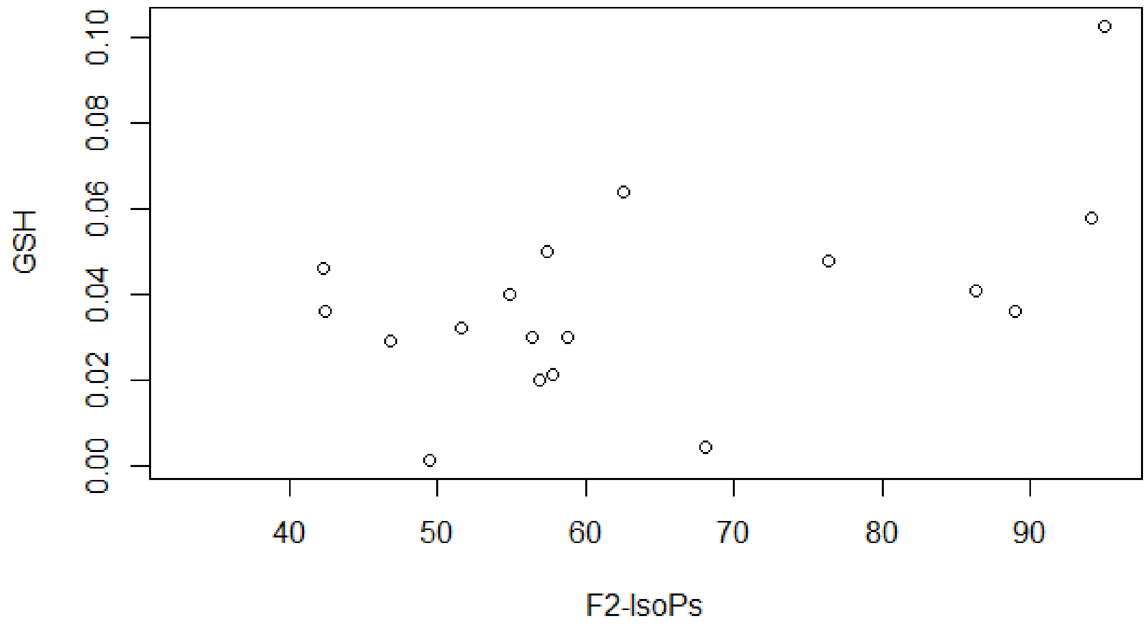
GSH
0.00
0.02
0.04
0.06
0.08
0.10
40
50
60
70
80
90
F2-IsoPs

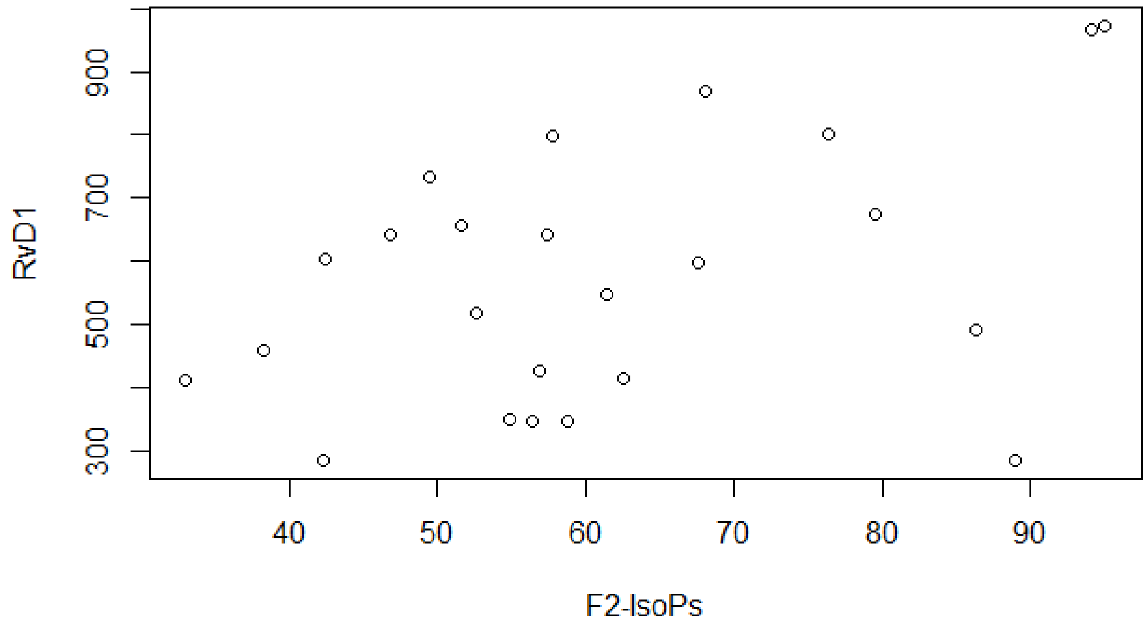

Scatter plots of pairs of variables giving rise to Pearson correlation coefficient significantly different from zero considering the group of women with age less than 38

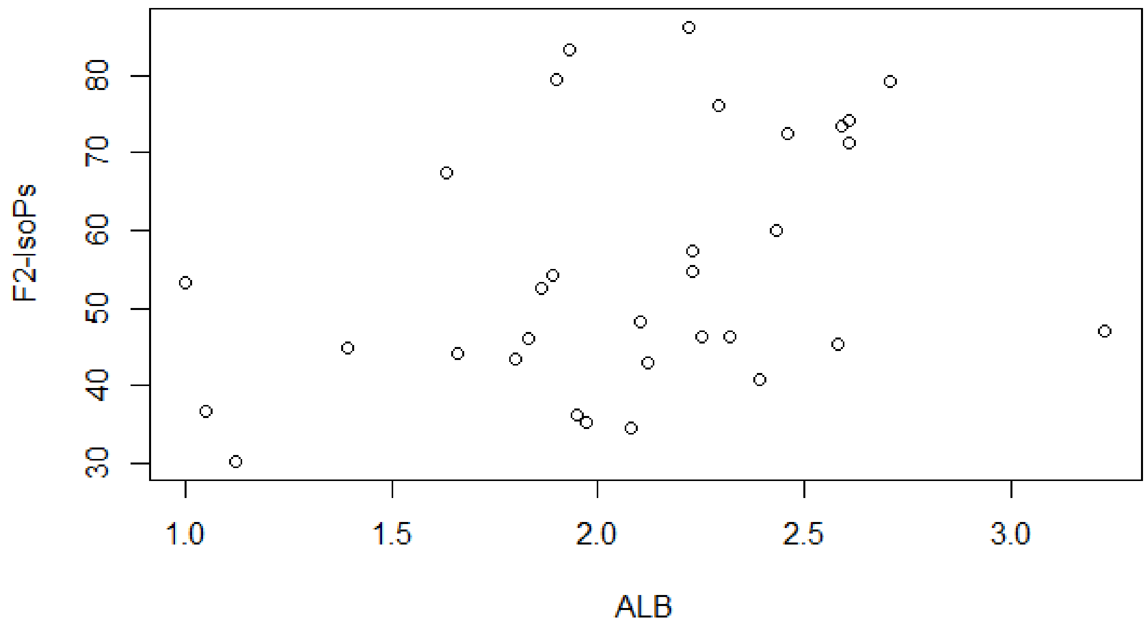

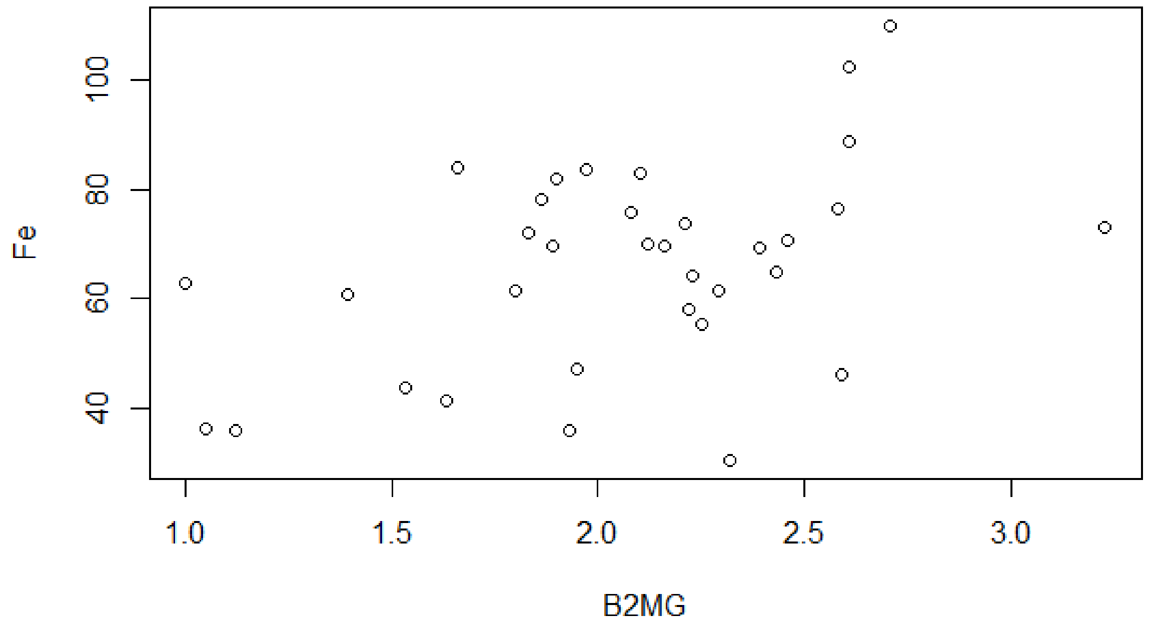
Fe
100
80
60
40
1.0
1.5
2.0
2.5
3.0
B2MG

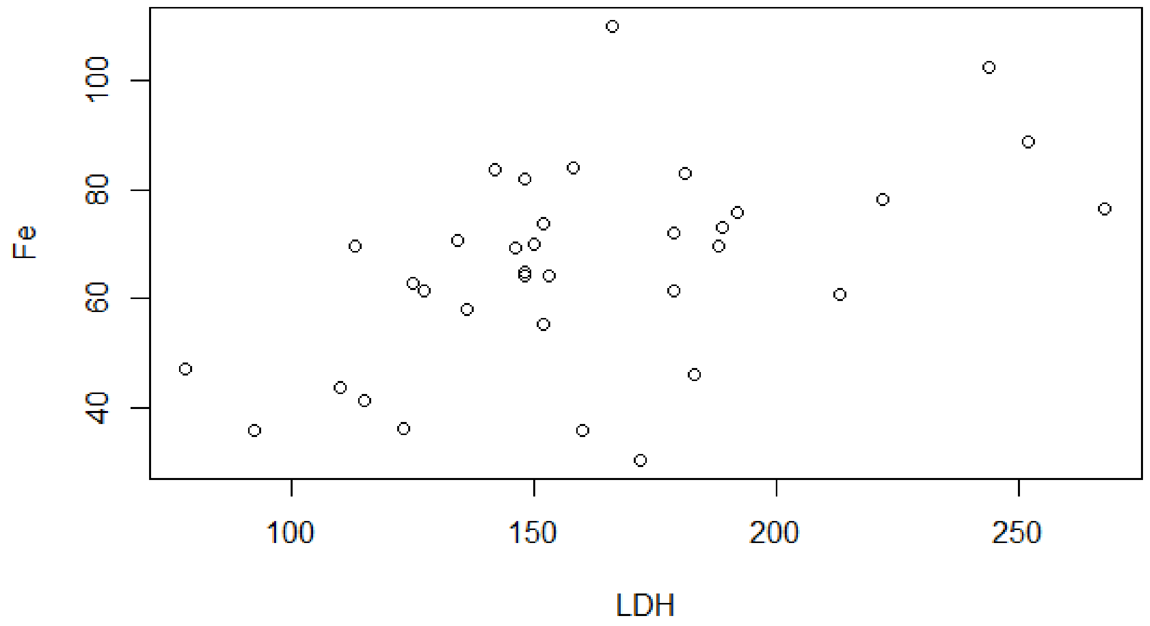
Fe
100
80
60
40
100
150
200
250
LDH

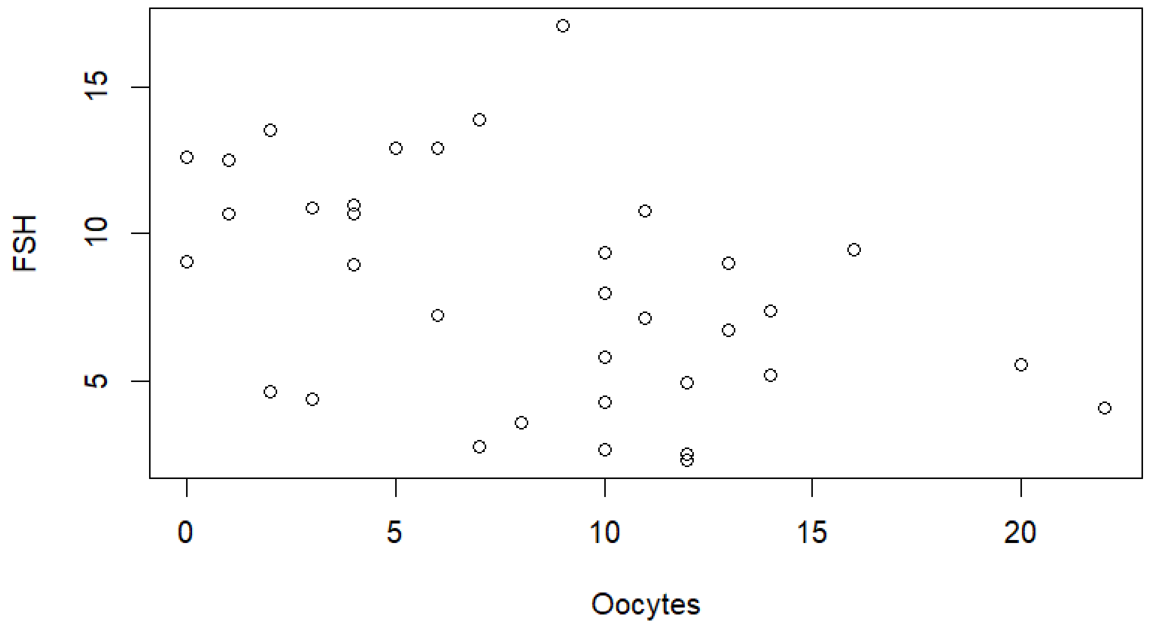

FSH
15
10
5
0
5
10
15
20
Oocytes

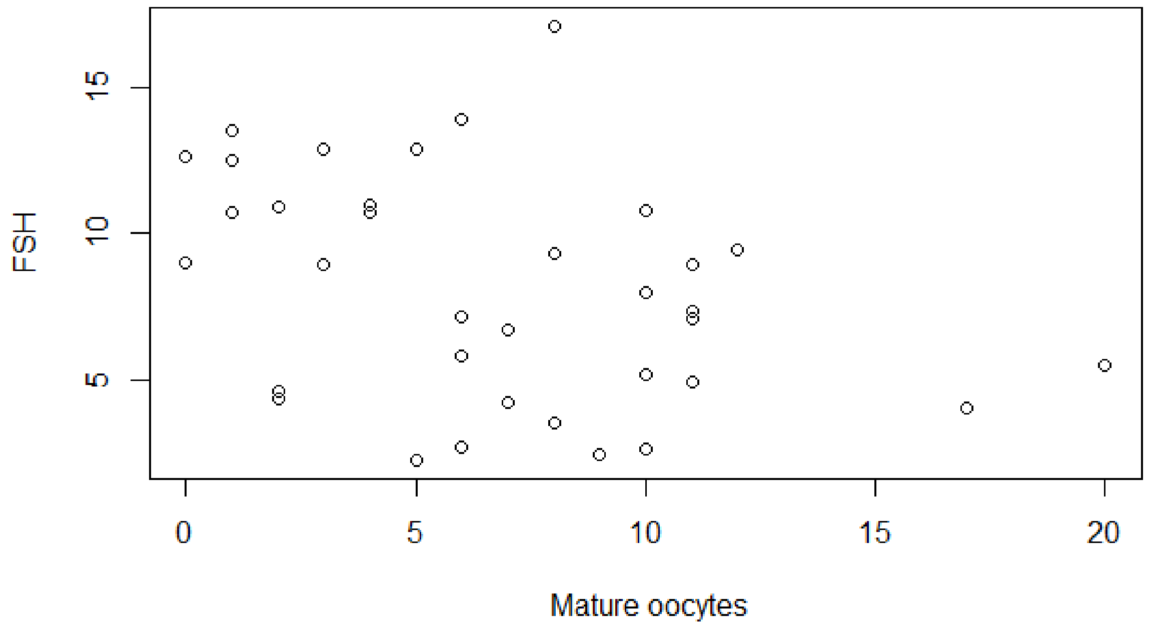

FSH
15
10
5
0
5
10
15
20
Mature oocytes

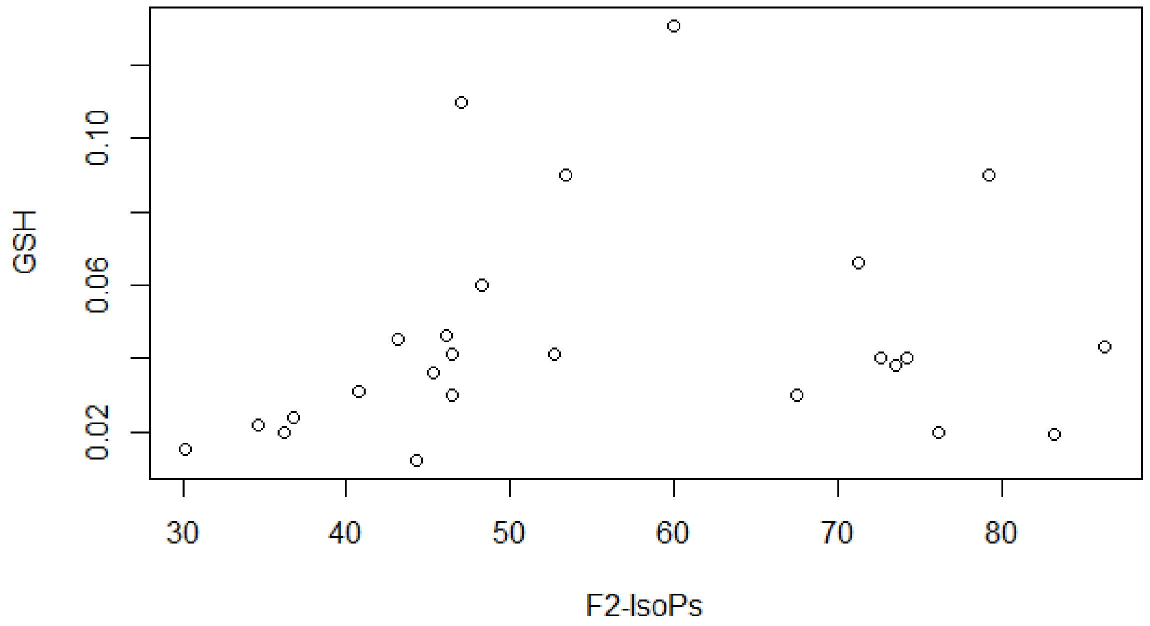
GSH
0.02
0.06
0.10
30
40
50
60
70
80
F2-IsoPs

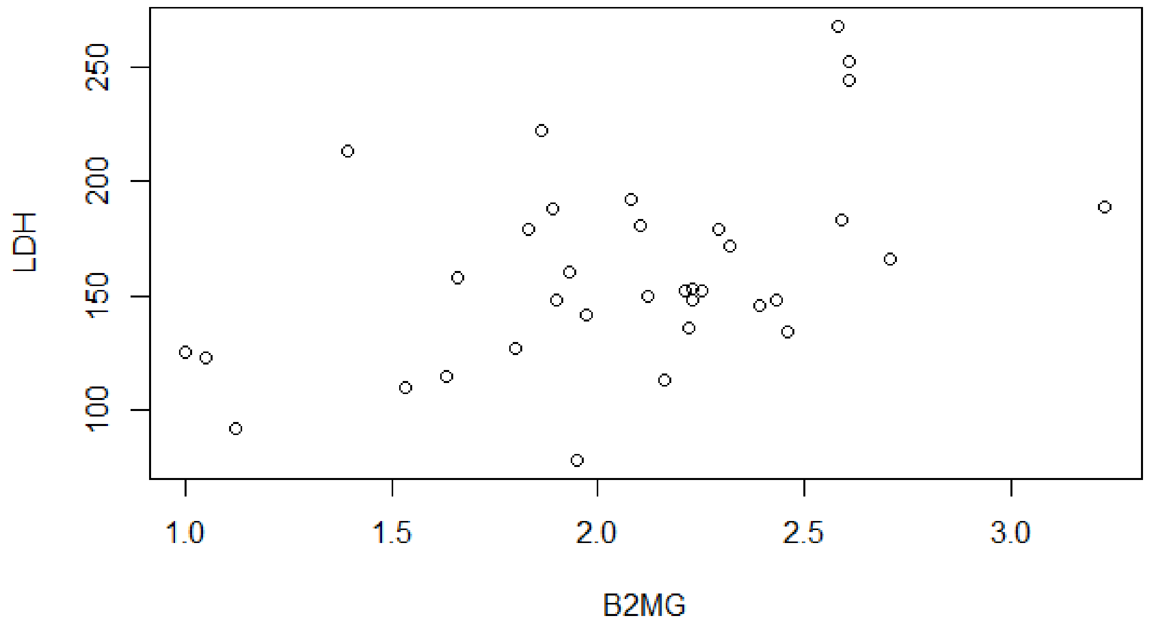
LDH
100
150
200
250
1.0
1.5
2.0
2.5
3.0
B2MG

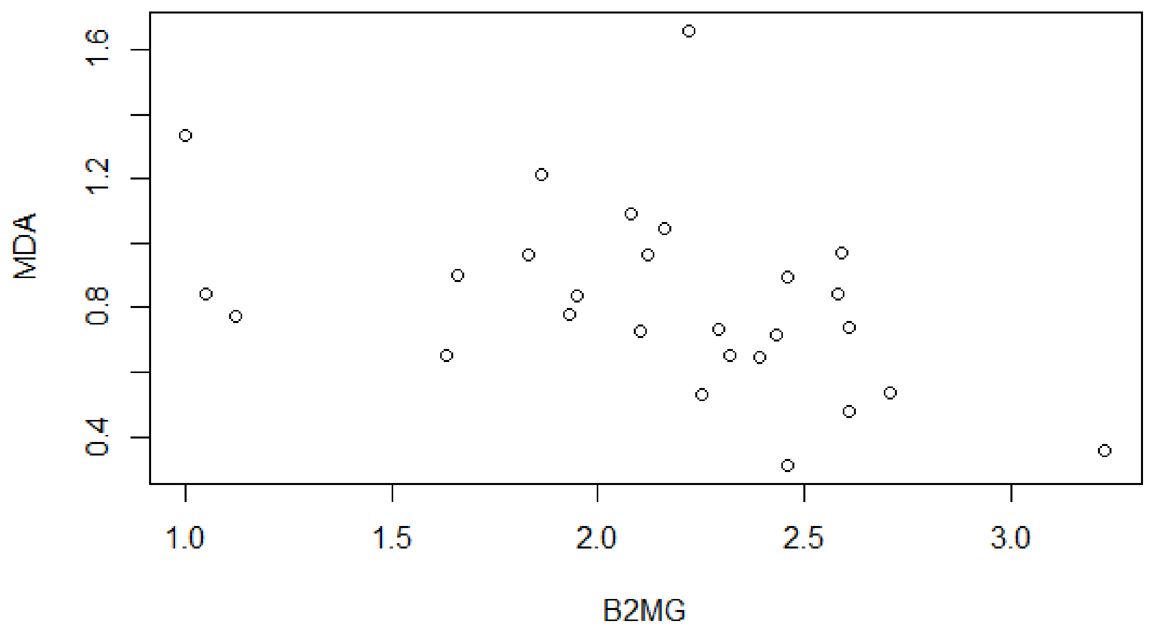
MDA
1.6
1.2
0.8
0.4
1.0
1.5
2.0
2.5
3.0
B2MG

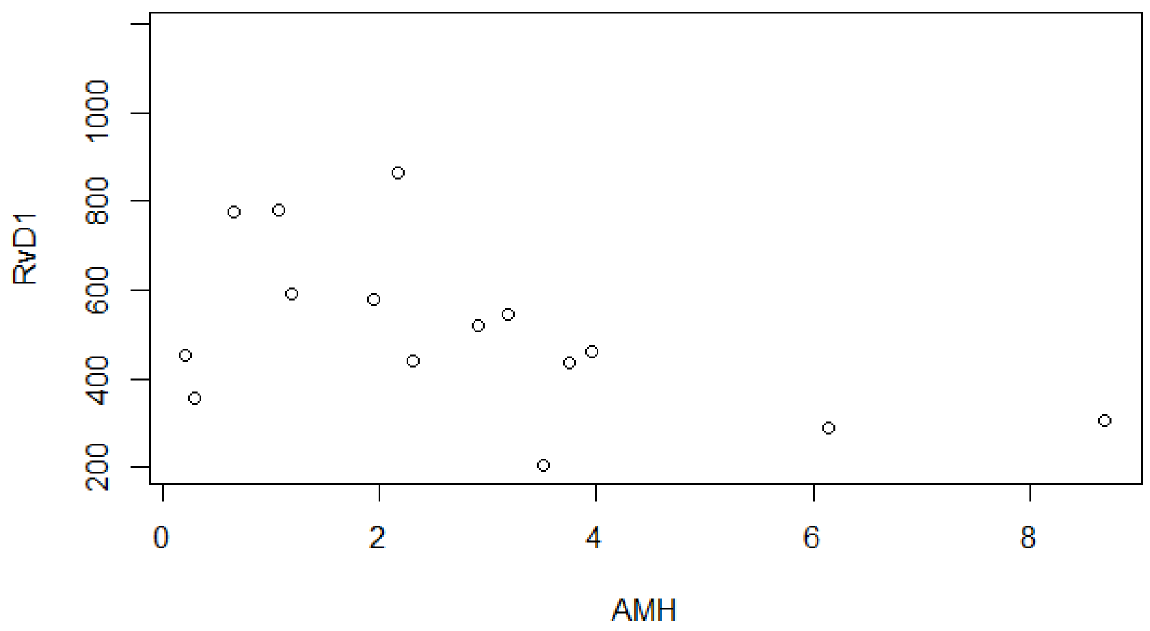
RvD1
1000
800
600
400
200
0
2
4
6
8
AMH

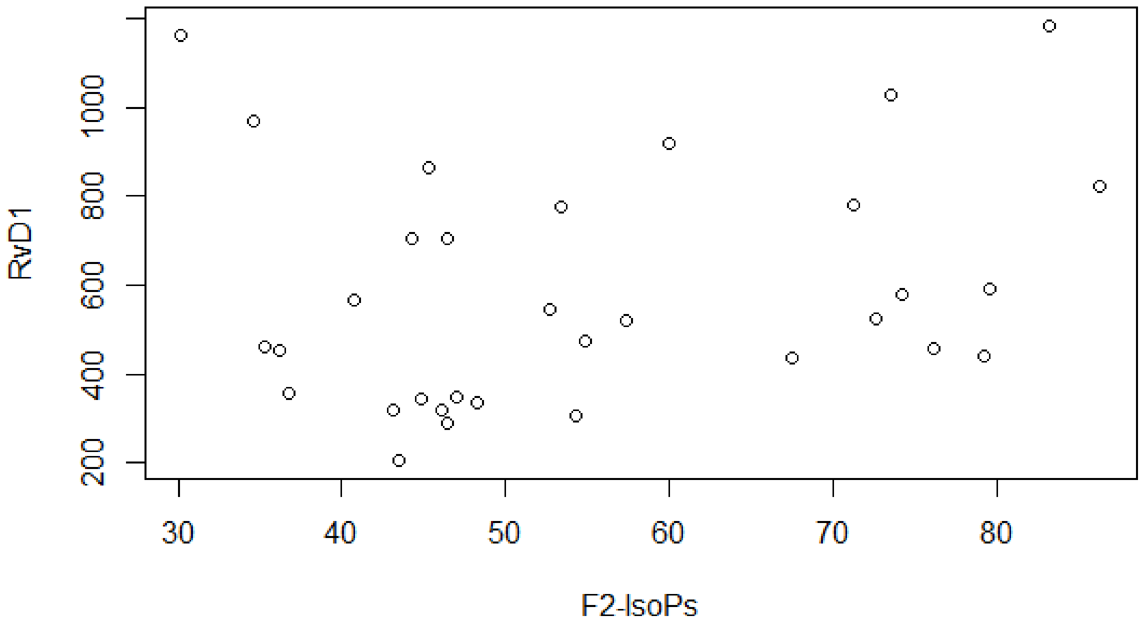

RvD1
200
400
600
800
1000
30
40
50
60
70
80
F2-IsoPs

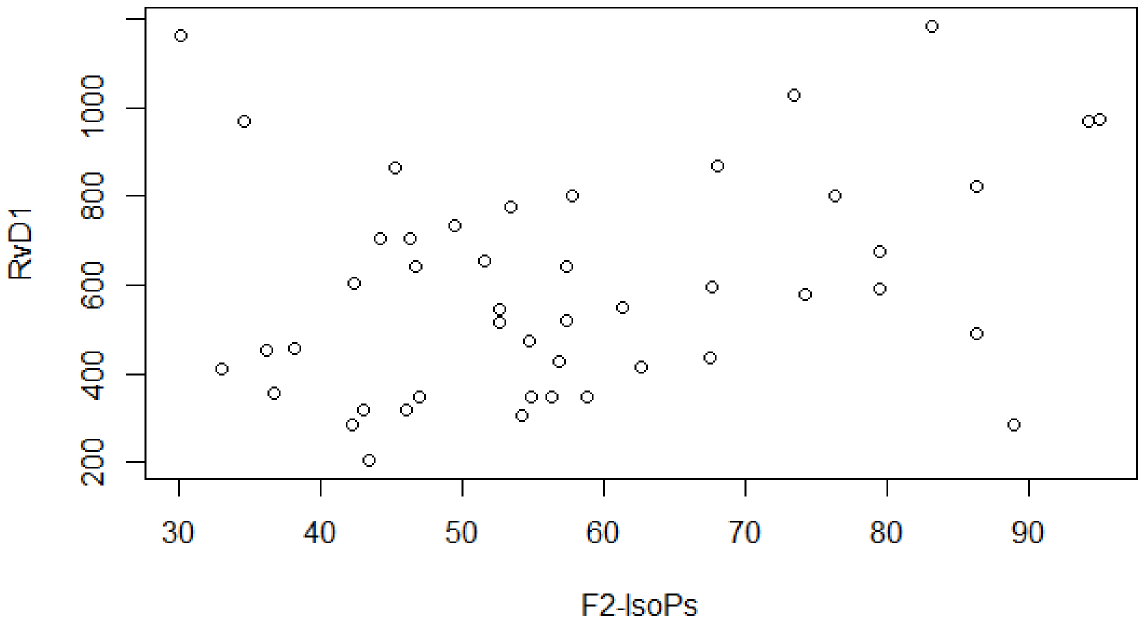

RvD1
200
400
600
800
1000
30
40
50
60
70
80
90
F2-IsoPs

Scatter plots of pairs of variables giving rise to Pearson correlation coefficient significantly different from zero considering the group of women with positive outcome for ART

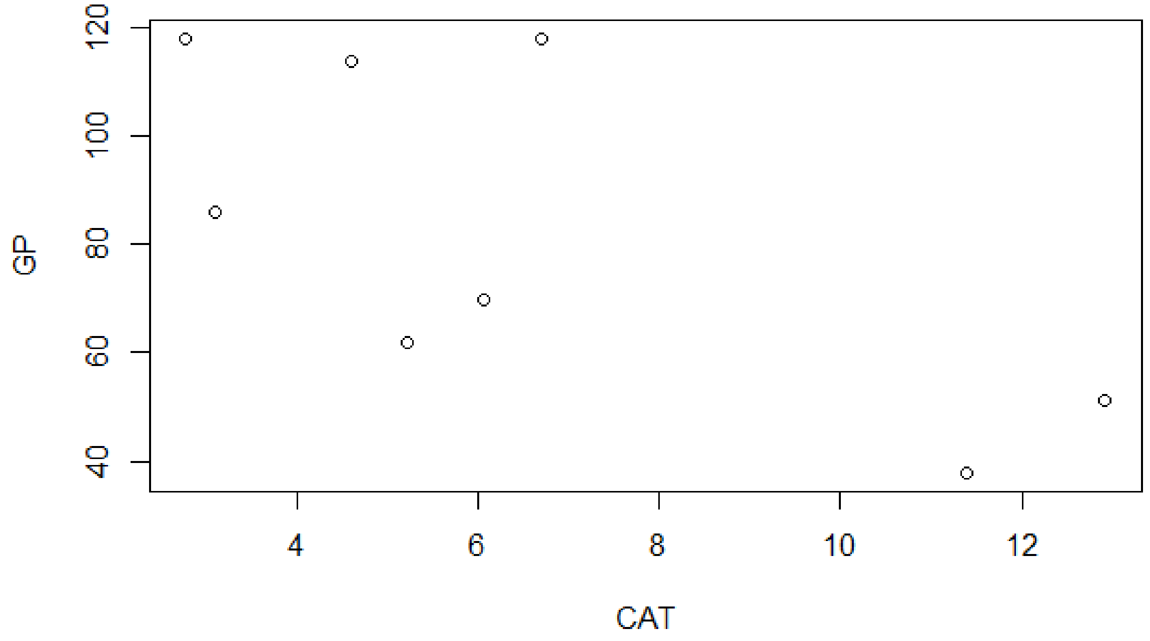

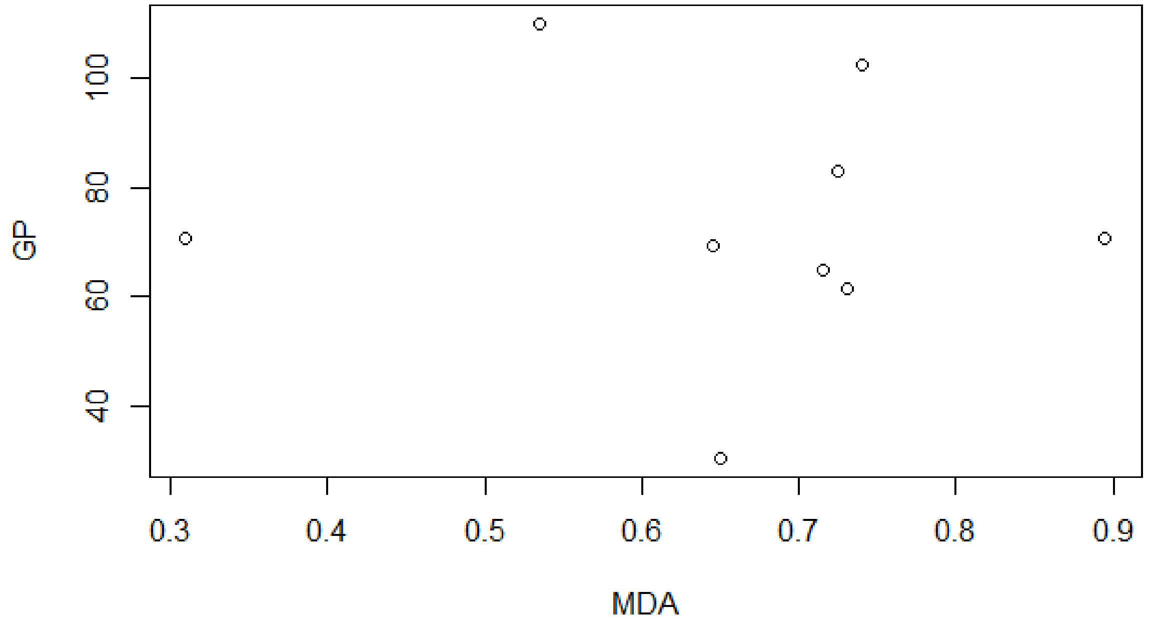

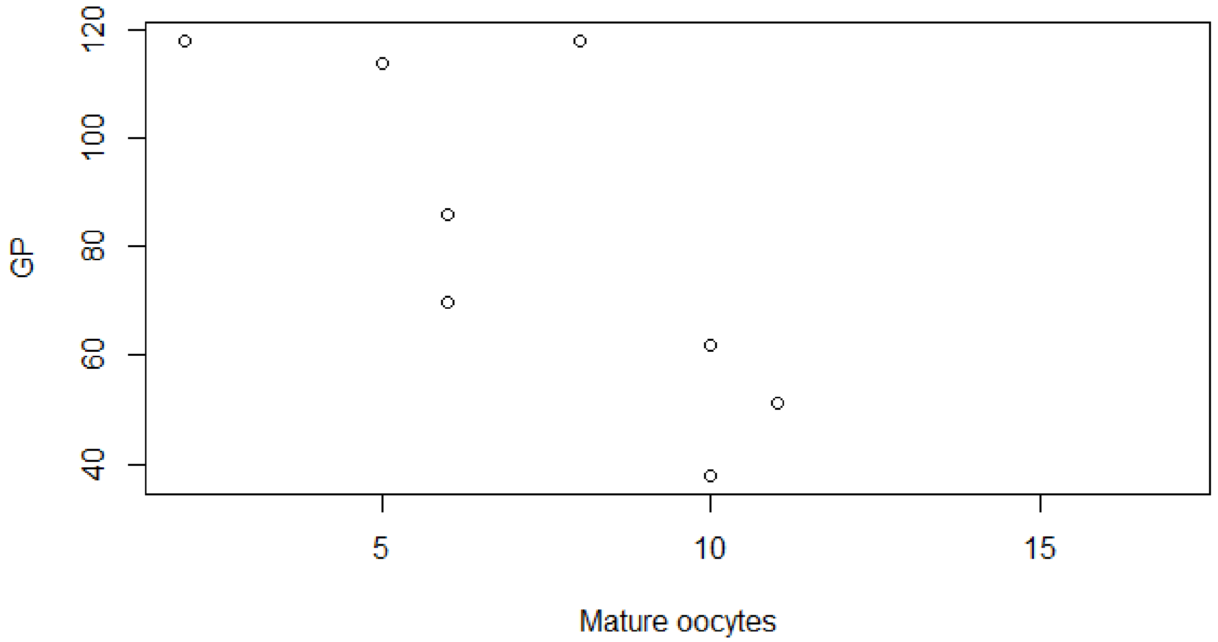

GP
120
100
80
60
40
5
10
15
Mature oocytes

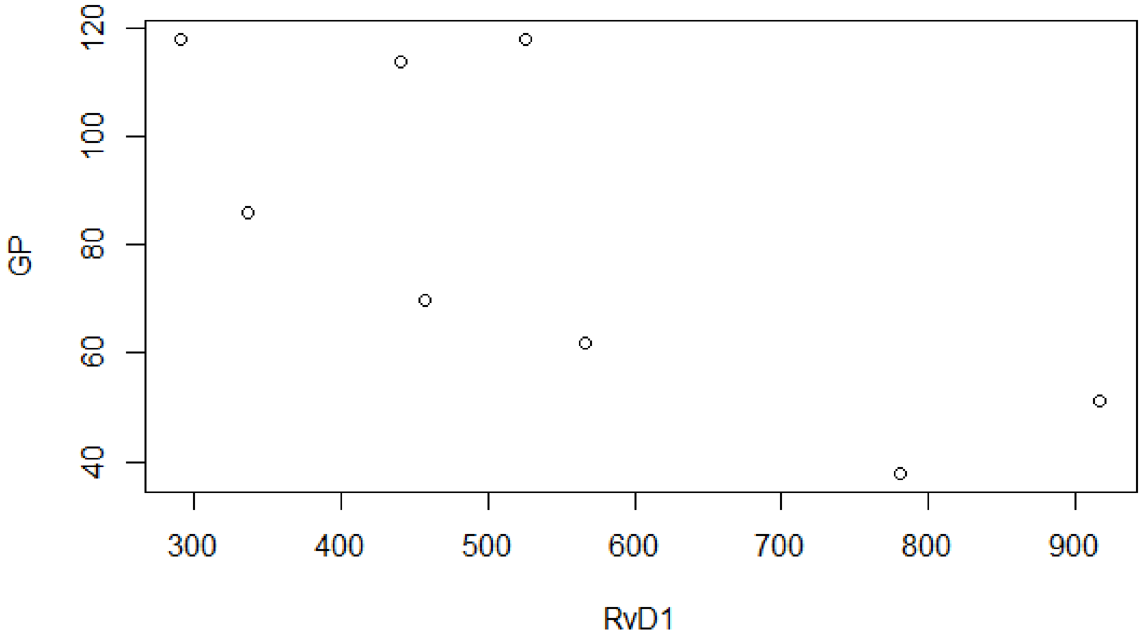

GP
120
100
80
60
40
300
400
500
600
700
800
900
RvD1

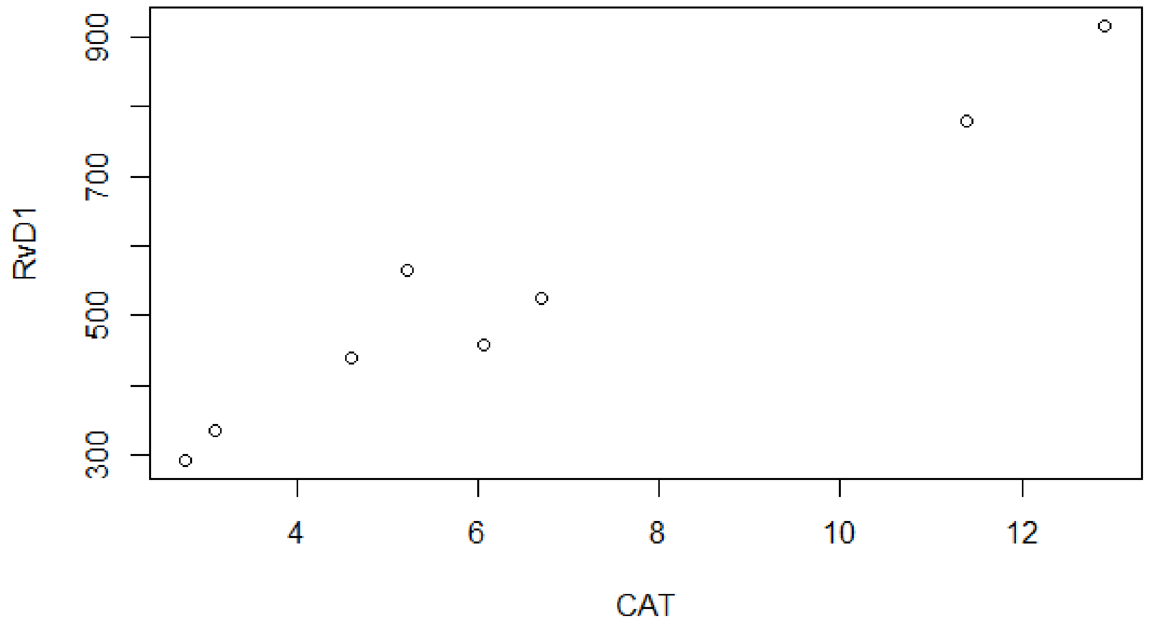

Scatter plots of the pair of variables giving rise to Pearson correlation coefficient significantly different from zero considering the group of women with negative outcome for ART

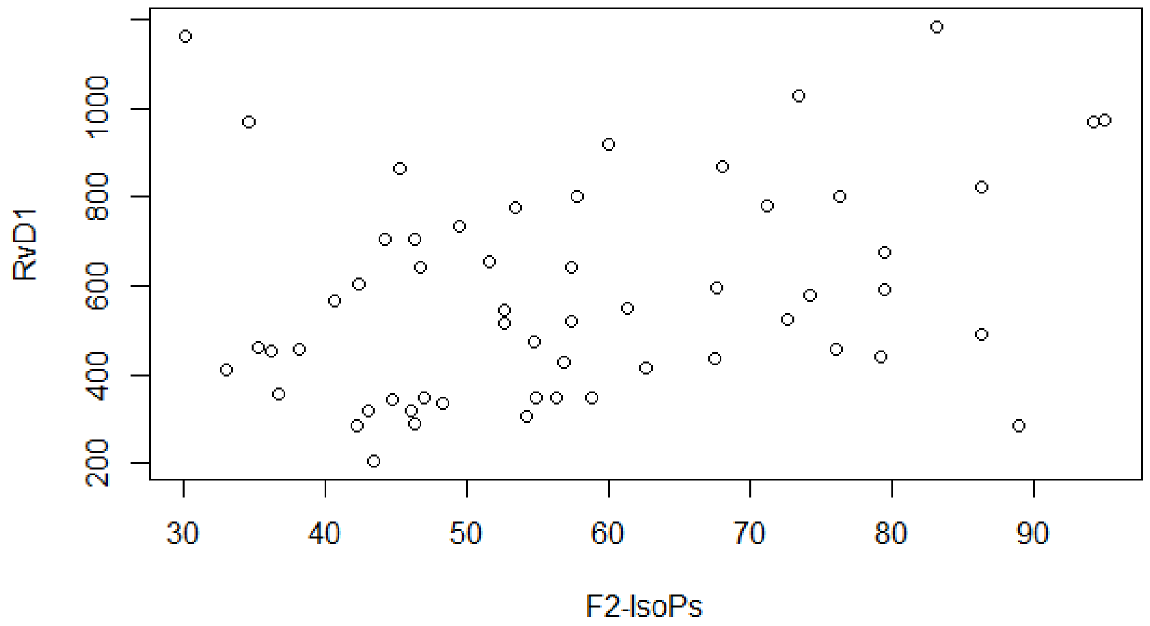

Supplement: Supplementary file 1 [file ijms-24-02589-s001.zip › ijms-2095375-supplementary.pdf]
